# Genome sequence of *Anoxybacillus ayderensis* AB04^T^ isolated from the Ayder hot spring in Turkey

**DOI:** 10.1186/s40793-015-0065-2

**Published:** 2015-09-26

**Authors:** Ali Osman Belduz, Sabriye Canakci, Kok-Gan Chan, Ummirul Mukminin Kahar, Chia Sing Chan, Amira Suriaty Yaakop, Kian Mau Goh

**Affiliations:** Faculty of Sciences, Department of Biology, Karadeniz Technical University, 61080 Trabzon, Turkey; Division of Genetics and Molecular Biology, Institute of Biological Sciences, Faculty of Science, University of Malaya, 50603 Kuala Lumpur, Malaysia; Faculty of Biosciences and Medical Engineering, Universiti Teknologi Malaysia, 81310 Skudai, Johor Malaysia

**Keywords:** *Anoxybacillus*, *Bacillaceae*, *Bacillus*, *Geobacillus*, Glycoside hydrolase, Thermophile

## Abstract

Species of *Anoxybacillus* are thermophiles and, therefore, their enzymes are suitable for many biotechnological applications. *Anoxybacillus ayderensis* AB04^T^ (= NCIMB 13972^T^ = NCCB 100050^T^) was isolated from the Ayder hot spring in Rize, Turkey, and is one of the earliest described *Anoxybacillus* type strains. The present work reports the cellular features of *A. ayderensis* AB04^T^, together with a high-quality draft genome sequence and its annotation. The genome is 2,832,347 bp long (74 contigs) and contains 2,895 protein-coding sequences and 103 RNA genes including 14 rRNAs, 88 tRNAs, and 1 tmRNA. Based on the genome annotation of strain AB04^T^, we identified genes encoding various glycoside hydrolases that are important for carbohydrate-related industries, which we compared with those of other, sequenced *Anoxybacillus* spp. Insights into under-explored industrially applicable enzymes and the possible applications of strain AB04^T^ were also described.

## Introduction

The family *Bacillaceae* [1, 2] is one of the largest bacterial families and currently consists of 57 genera [3]. The *Bacillaceae* are either rod-shaped (bacilli) or spherical (cocci) Gram-positive bacteria, the majority of which produce endospores [4]. *Anoxybacillus* [5, 6] is one of the genera within the *Bacillaceae* [1, 2], classified within the phylum *Firmicutes* [7], class *Bacilli* [8, 9], and order *Bacillales* [1, 10].

*Anoxybacillus* spp. are alkalo-thermophiles with optimum growth at temperatures between 50 °C and 65 °C and at pH 5.6–9.7 [4]. Most of the *Anoxybacillus* spp. are found in hot springs [4], but *Anoxybacillus* has also been found in animal manure [5], contaminated diary and meat products [4], animals (i.e., fish gut) [4], insects (i.e., glassy-winged sharpshooter and spiraling whitefly) [11], and plants (i.e., Indian mulberry) [11]. To date, a total of 22 species and two subspecies of *Anoxybacillus* have been described [4, 12, 13].

Almost all members of the *Bacillaceae* are excellent industrial enzyme producers [4, 14, 15]. Members of the genus *Anoxybacillus* exhibit the additional advantage of thermostability compared to the mesophilic *Bacillaceae*. It has been reported that enzymes from *Anoxybacillus* spp. can degrade various substrates such as starches, cellulose, fats, and proteins [4]. Many carbohydrase-encoding genes have been identified in *Anoxybacillus* spp. genomes, and some of the well-studied starch-degrading enzymes are α-amylase [16], pullulanase [17], amylopullulanase [18], CDase [19], and xylose-isomerase [20]. In addition, xylanolytic enzymes such as xylanase [21] and α-L-arabinofuranosidase [22] have been characterized from *Anoxybacillus* spp. Apart from their hydrolytic capabilities, *Anoxybacillus* spp. have been proposed as agents for bioremediation of Hg^2+^, Cr^2+^, Al^3+^, As^3+^ ions [4, 23–25], and nitrogen oxide [26], and as possible candidates for biohydrogen production [4].

Among the members of the family *Bacillaceae*, intensive genome sequencing efforts have been undertaken for *Geobacillus* [27] (>80 projects) and *Bacillus* [1, 28] (>1,500 projects), which have been registered in the NCBI BioProject database. In contrast, genomic studies on *Anoxybacillus* are rather limited, with only 16 registered projects. At present, the genome of *Anoxybacillus flavithermus* WK1 is the only completely sequenced genome (BioProject accession number PRJNA59135) among the *Anoxybacillus* spp. [5, 29]. Draft genome sequences are available for *Anoxybacillus ayderensis* AB04^T^ (PRJNA258494; this study) [30], *Anoxybacillus* sp. BCO1 (PRJNA261743) [31, 32], *Anoxybacillus thermarum* AF/04^T^ (PRJNA260786) [33–35], *Anoxybacillus gonensis* G2^T^ (PRJNA264351) [36], *Anoxybacillus* sp. ATCC BAA-2555 (PRJNA260743), *Anoxybacillus* sp. KU2-6(11) (PRJNA258246), *Anoxybacillus tepidamans* PS2 (PRJNA214279) [37], *A. flavithermus* 25 (PRJNA258119) [5, 38], *A. flavithermus* AK1 (PRJNA190633) [5, 39], *Anoxybacillus kamchatkensis* G10 (PRJNA170961) [40–42], *A. flavithermus* Kn10 (PRJDB1085) [5, 43], *A. flavithermus* TNO-09.006 (PRJNA169174) [5, 44], *Anoxybacillus* sp. SK3-4 (PRJNA174378) [45, 46], *Anoxybacillus* sp. DT3-1 (PRJNA182115) [45, 46], and *A. flavithermus* subsp*. yunnanensis* E13^T^ (PRJNA213809) [35, 47, 48]. Therefore, the genomic study of *Anoxybacillus* spp. is essential not only to fully understand their biochemical networks, but also to discover their potential applicability in industrial processes.

In the present report, we describe the cellular features of *A. ayderensis* AB04^T^ and we present a high-quality annotated draft genome of strain AB04^T^. Additionally, we provide a comparative analysis of the GHs of strain AB04^T^ and other sequenced *Anoxybacillus* spp. In addition, we discuss the presence of other under-explored industrial enzymes and the potential applications of the bacterium.

## Organism information

### Classification and features

*A. ayderensis* AB04^T^ (= NCIMB 13972^T^ = NCCB 100050^T^) was isolated from mud and water samples from the Ayder hot spring located in the province of Rize in Turkey [30]. Microscopic examination revealed that colonies of strain AB04^T^ were cream-colored, regular in shape with round edges, and 1–2 mm in diameter.

**Fig. 1:**
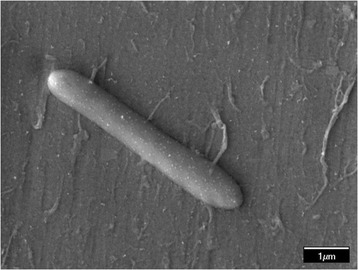
FESEM micrograph of *A. ayderensis* AB04^T^. The micrograph was captured using FESEM (JEOL JSM-6701 F, Tokyo, Japan) operating at 5.0 kV at a magnification of 15,000 ×

Phenotypic analysis revealed that strain AB04^T^ is a Gram-positive, rod-shaped, motile, and spore-forming bacterium [30]. It is a facultative anaerobe, moderate thermophile that grows well at 30–70 °C (optimum 50 °C) and at pH 6.0–11.0 (optimum pH 7.5–8.5) (Table 1). FESEM showed that cells of the strain AB04^T^ were 0.7–0.8 × 3.5–5.0 μm in size (Fig. 1). The strain gave positive responses for catalase and oxidase activity, and was able to reduce nitrate to nitrite. Strain AB04^T^ was capable of utilizing a wide range of carbon sources including starch, gelatin, d-glucose, d-raffinose, d-sucrose, d-xylose, d-fructose, l-arabinose, maltose, and d-mannose. The strain grew optimally in the presence of 1.5 % (w/v) NaCl, but it was able to grow in the absence of NaCl. Growth was inhibited in the presence of ampicillin (25 μg/ml), streptomycin sulphate (25 μg/ml), tetracycline (12.5 μg/ml), gentamicin (10 μg/ml), and kanamycin (10 μg/ml). The FAME profile showed that the major fatty acid in AB04^T^ is C_15:0_iso (48.17 %), followed by C_17:0_ iso (20.62 %), C_17:0_ anteiso (9.22 %), C_16:0_ (9.10), C_16:0_ iso (7.47 %), C_15:0_ anteiso (3.58 %), C_14:0_ (1.02 %), and C_15:0_ (0.83 %) [30].

**Table 1:** Classification and general features of *A. ayderensis* AB04^T^ [74]

MIGS ID	Property	Term	Evidence code^a^
	Classification	Domain *Bacteria*	TAS [75]
		Phylum *Firmucutes*	TAS [7]
		*Class Bacilli*	TAS [8, 9]
		Order *Bacillales*	TAS [1, 10]
		Family *Bacillaceae*	TAS [1, 2]
		Genus *Anoxybacillus*	TAS [5, 6]
		Species *Anoxybacillus ayderensis*	TAS [30]
		Type strain: AB04^T^ (NCIMB 13972^T^, NCCB 100050^T^)	TAS [30]
	Gram stain	Positive	TAS [30]
	Cell shape	Rod	TAS [30]
	Motility	Motile	TAS [30]
	Sporulation	Terminal, spherical endospore	TAS [30]
	Temperature range	30-70 °C	TAS [30]
	Optimum temperature	50 °C	TAS [30]
	pH range; Optimum	6.0-11.0; 7.5-8.5	TAS [30]
	Carbon source	Carbohydrates	TAS [30]
MIGS-6	Habitat	Hot spring	TAS [30]
MIGS-6.3	Salinity	Optimum at 1.5 % NaCl (w/v)	TAS [30]
MIGS-22	Oxygen requirement	Facultative anaerobe	TAS [30]
MIGS-15	Biotic relationship	Free-living	TAS [30]
MIGS-14	Pathogenicity	Non-pathogenic	TAS [30]
MIGS-4	Geographic location	Ayder hot spring, Rize, Turkey	IDA
MIGS-5	Sample collection	January 1995	IDA
MIGS-4.1	Latitude	40°57’N	IDA
MIGS-4.2	Longitude	41°05’E	IDA
MIGS-4.4	Altitude	1350 m above sea level	IDA

^a^Evidence codes – *IDA* Inferred from Direct Assay, *TAS* Traceable Author Statement (i.e., a direct report exists in the literature), *NAS* Non-traceable Author Statement (i.e., not directly observed for the living, isolated sample, but based on a generally accepted property for the species, or anecdotal evidence). These evidence codes are from the Gene Ontology project [76]

**Fig. 2:**
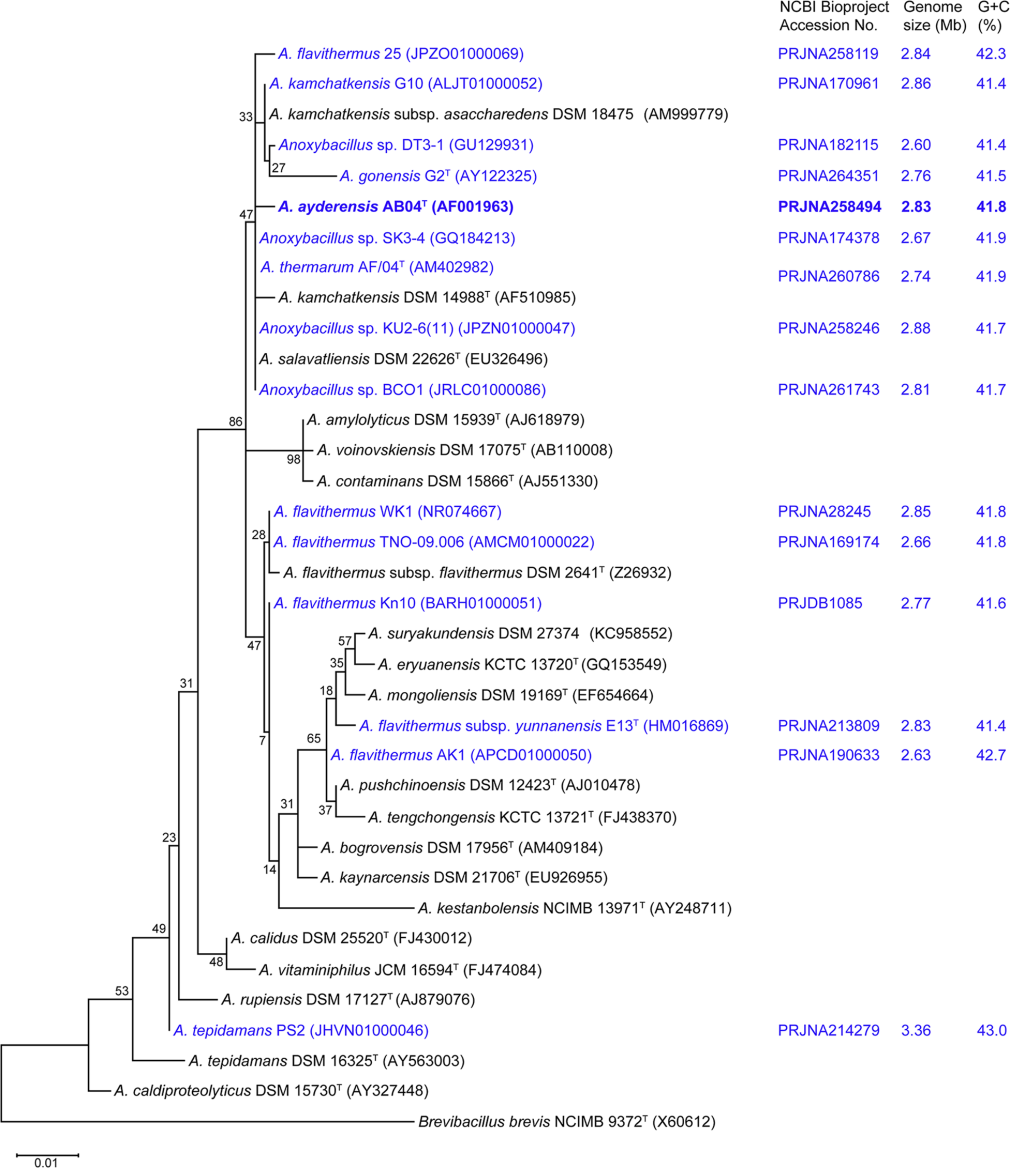
Phylogenetic tree based on 16S rRNA gene sequences showing the relationship between *A. ayderensis* AB04^T^ and representative *Anoxybacillus* spp. The 16S rRNA accession number for each strain is shown in brackets. The 16S rRNA sequences were aligned using ClustalW and the tree was constructed using the ML method with 1000 bootstrap replicates embedded in the MEGA6.0 package [49]. The scale bar represents 0.01 nucleotide substitutions per position. *Brevibacillus brevis* NCIMB 9372^T^ [77] was used as an out-group. Type strains are indicated with a superscript T. Published genomes are indicated in blue

The 16S rRNA-based phylogenetic tree constructed using MEGA6.0 [49] showed that strain AB04^T^ clusters together with *Anoxybacillus* sp. SK3-4 [45, 46] and *A. thermarum* AF/04^T^ [33–35] (Fig. 2). Pairwise 16S rRNA sequence similarities among the strains were determined using the EzTaxon server [50], revealing that AB04^T^ shares 99.6 % and 99.2 % similarity with *Anoxybacillus* sp. SK3-4 [45, 46] and *A. thermarum* AF/04^T^ [33–35], respectively.

## Genome sequencing information

### Genome project history

Genomic studies on the genus *Anoxybacillus* are relatively limited [45]. Hence, the findings of the genomic study on *A. ayderensis* AB04^T^ presented in this study are important because they contribute to the body knowledge of the *Anoxybacillus* genomes. This whole-genome shotgun project has been deposited at DDBJ/EMBL/GenBank under the accession number JXTG00000000. The NCBI BioProject accession number is PRJNA258494. The GOLD Project ID for strain AB04^T^ is Gp0026071. Table 2 presents the project information and its association with MIGS version 2.0 compliance.

**Table 2:** Project information

MIGS ID	Property	Term
MIGS-31	Finishing quality	High-quality draft
MIGS-28	Libraries used	Illumina Paired-End library
MIGS-29	Sequencing platforms	Illumina MiSeq
MIGS-31.2	Fold coverage	239 ×
MIGS-30	Assemblers	IDBA-UD 1.0.9
MIGS-32	Gene calling method	Prodigal 2.60
	Locus Tag	JV16
	Genbank ID	JXTG00000000
	Genome Data of Release	February 9, 2015
	GOLD ID	Gp0026071
	BIOPROJECT	PRJNA258494
MIGS-13	Source Material Identifier	NCIMB 13972^T^
	Project relevance	Biotechnology

### Growth conditions and genomic DNA preparation

*A. ayderensis* AB04^T^ was plated on Nutrient Agar (pH 7.5) and incubated at 50 °C for 18 h. A single colony was transferred into Nutrient Broth (pH 7.5) and incubated at 50 °C with rotary shaking at 200 rpm for 18 h. The cells were harvested by centrifugation at 10,000 × *g* for 5 min using a Microfuge^®^ 16 centrifuge (Beckman Coulter, Brea, CA, USA). Genomic DNA was extracted using a Qiagen DNeasy Blood & Tissue Kit (Qiagen, Hilden, Germany) according to the manufacturer’s protocol. The purity, quality, and concentration of the genomic DNA were determined using a 6 % (w/v) agarose gel, NanoDrop 1000 spectrophotometer (Thermo Scientific, Wilmington, DE, USA), and Qubit 2.0 fluorometer (Invitrogen, Merelbeke, Belgium).

### Genome sequencing and assembly

The genome of *A. ayderensis* AB04^T^ was sequenced using the Illumina MiSeq sequencing platform (Illumina, San Diego, CA, USA) with 300-bp paired-end reads. The adapter sequences were removed and low quality regions and reads were filtered out using Trimmomatic [51] (Phred score = 25 (Q25), sliding window = 4 bp, leading and trailing qualities = 3, and minimum read length = 36 bp), Scythe (UC Davis Bioinformatics Core, Davis, DA, USA) (prior contamination rate = 0.3, minimum match length argument = 5, and minimum sequence to keep after trimming = 36 bp), and String Graph Assembler (SGA) [52] (*k-mer* threshold = 3, *k-mer* rounds = 10, and read error correction = 0.04). Next, the reads were subjected to *de novo* genome assembly using IDBA-UD 1.0.9 [53] (*k*_*min*_ = 35).

### Genome annotation

Genes, tRNAs and tmRNAs, and rRNAs were predicted with Prodigal [54], ARAGORN [55], and RNAmmer [56], respectively. For functional annotation, the predicted coding sequences were translated and used to search for the closest matches in the NCBI non-redundant database and the UniProt [57], TIGRFAM [58], Pfam [59], CRISPRfinder [60], PRIAM [61], KEGG [62], COG [63], and InterProScan 5 [64] databases. The GHs were identified and verified using the dbCAN CAZy [65], NCBI BLASTp, and InterProScan 5 [64] databases. Genome comparison was done by the ANI function in the EzTaxon-e database [66].

## Genome properties

**Fig. 3:**
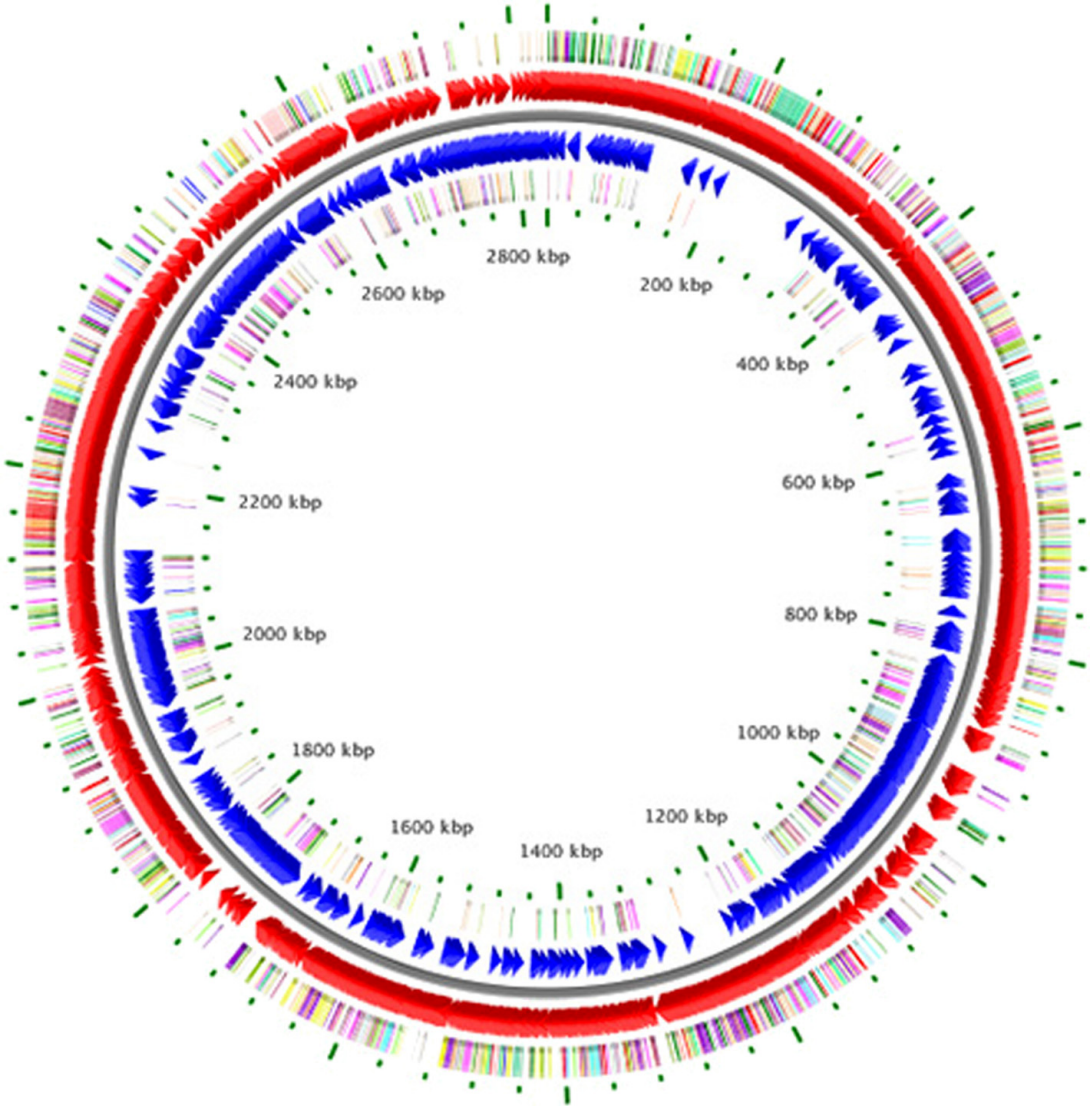
A graphical circular map of the *A. ayderensis* AB04^T^ genome. From outside to the center: genes on the forward strand (colored by COG categories), genes on forward strand (red), genes on reverse strand (blue) and genes on the reverse strand (colored by COG categories)

The overall genome coverage was approximately 239-fold. The draft genome was assembled into 74 contigs with a total length of 2,832,347 bp and a G + C content of 41.8 % (Fig. 3 and Table 3). The longest and shortest contigs were 448,584 bp and 606 bp, respectively. The mean length of the contigs was 38,275 bp and the N50 contig length was 112,260 bp. We did not detect any additional DNA elements. The genome consisted of 2,998 predicted genes, of which 2,895 were protein-coding sequences and 103 were RNA genes including 14 rRNAs, 88 tRNAs, and 1 tmRNA. A total of 235 (8.1 %) genes were assigned a putative function. The remaining annotated genes (1023; 35.3 %) were hypothetical proteins. The properties and the statistics of the genome are summarized in Table 3. The distribution of genes into COGs and KEGG functional categories is presented in Table 4 and Fig. 3.

**Table 3:** Genome statistics

Attribute	Value	% of Total^a^
Genome size (bp)	2,832,347	100.00
DNA coding (bp)	2,517,744	88.89
DNA G + C (bp)		41.83
DNA scaffolds	74	100.00
Total genes	2,998	100.00
Protein coding genes	2,895	96.56
RNA genes	103	3.44
Pseudo genes	not determined	not determined
Genes in internal clusters	not determined	not determined
Genes with function prediction	1,637	54.60
Genes assigned to COGs	2,349	78.35
Genes with Pfam domains	2,158	71.98
Genes with signal peptides	103	3.44
Genes with transmembrane helices	674	23.28
Number of CRISPR candidates	3	
Confirmed CRISPR(s)	1	
Unconfirmed CRISPR(s)	2	

^a^The total is based on either the size of the genome in base pairs or the total number of protein coding genes in the annotated genome

**Table 4:** Number of genes associated with general COG functional categories

Code	Value	% age^a^	Description
J	153	5.10	Translation, ribosomal structure and biogenesis
A	1	0.03	RNA processing and modification
K	169	5.64	Transcription
L	165	5.50	Replication, recombination and repair
B	1	0.03	Chromatin structure and dynamics
D	38	1.27	Cell cycle control, Cell division, chromosome partitioning
V	27	0.90	Defense mechanisms
T	162	5.40	Signal transduction mechanisms
M	117	3.90	Cell wall/membrane biogenesis
N	80	2.67	Cell motility
U	53	1.77	Intracellular trafficking and secretion
O	99	3.30	Posttranslational modification, protein turnover, chaperones
C	145	4.84	Energy production and conversion
G	169	5.64	Carbohydrate transport and metabolism
E	234	7.81	Amino acid transport and metabolism
F	71	2.37	Nucleotide transport and metabolism
H	120	4.00	Coenzyme transport and metabolism
I	81	2.70	Lipid transport and metabolism
P	140	4.67	Inorganic ion transport and metabolism
Q	29	0.97	Secondary metabolites biosynthesis, transport and catabolism
R	274	9.14	General function prediction only
S	261	8.71	Function unknown
-	409	13.64	Not in COGs

^a^The total is based on the total number of protein coding genes in the annotated genome

## Insights from the genome sequence

### Genome features of ***A. ayderensis*** AB04^T^ and other ***Anoxybacillus*** spp

The genome sizes of the currently sequenced *Anoxybacillus* spp. are shown in Fig. 2. Most of the reported *Anoxybacillus* draft genome sizes are between 2.60 and 2.86 Mb [31, 33, 38–40, 43–45, 47], and the completely sequenced *A. flavithermus* WK1 genome has a size of 2.85 Mb [29]. The incomplete genome sequence of *A. tepidamans* PS2 has a size of 3.36 Mb (Fig. 2), which is the largest *Anoxybacillus* genome sequenced to date [37]. However, cumulative information on the *Anoxybacillus* genomes (Fig. 2) indicates that *Anoxybacillus* has a smaller genome size than the closest genus, *Geobacillus* (~3.50 Mb) [27, 45]. The genomes of other genera within *Bacillaceae* such as *Bacillus* [1, 28] and *Lysinibacillus* [67] are at least 40 % larger than that of *Anoxybacillus* [5, 6, 45]. The average G + C content of the *Geobacillus* spp. genomes (~50.0 %) [27, 45] is slightly higher than that of the *A. ayderensis* [30] genome (Fig. 2), while most *Bacillus* genomes have less than 40 % G + C content [1, 28, 45].

Table 5 summarizes the pairwise ANI values of *Anoxybacillus* spp. [66]. *A. ayderensis* AB04^T^ showed the highest ANI of 97.6 % with *Anoxybacillus* sp. SK3-4 [46]. As this ANI value is greater than 95 % [68], *Anoxybacillus* sp. SK3-4 [45, 46] is likely to be a subspecies of *A. ayderensis* [30].

**Table 5:** Genomic comparison of *A. ayderensis* AB04^T^ and 15 other sequenced *Anoxybacillus* spp. using ANI [66]

	AB04^T^	WK1	E13^T^	SK3-4	DT3-1	TNO	G10	G2^T^	AF/04^T^	AK1	BCO1	KU2-6	Kn10	PS2	25
AB04^T^	100.00	87.9	87.3	97.6	94.5	87.3	94.3	94.3	94.7	85.7	97.5	89.6	88.2	72.4	97.6
WK1	87.9	100.00	88.4	88.0	88.1	91.8	88.2	88.2	87.8	84.8	87.7	89.8	95.0	72.5	87.5
E13^T^	87.3	88.3	100.00	87.3	87.2	88.3	86.9	87.1	86.9	85.2	87.1	89.9	89.1	72.3	87.1
SK3-4	97.5	88.1	87.2	100.00	94.0	87.5	93.7	93.9	94.2	85.8	96.9	89.5	88.3	72.5	96.9
DT3-1	94.6	88.0	87.2	94.1	100.00	87.0	98.5	98.6	94.1	85.3	94.4	89.8	88.0	72.4	94.1
TNO	87.5	91.8	88.4	87.7	87.1	100.00	87.1	87.0	87.3	87.5	87.4	88.6	92.5	72.5	87.3
G10	94.3	88.2	86.8	93.8	98.5	87.0	100.00	98.8	93.7	85.3	94.3	89.7	88.2	72.6	94.0
G2^T^	94.4	88.2	87.1	94.0	98.5	87.0	98.8	100.00	93.8	85.3	94.2	89.7	88.3	72.4	93.8
AF/04^T^	94.8	87.9	87.0	94.2	94.1	87.2	93.8	93.8	100.00	86.1	94.1	89.1	88.1	72.7	94.0
AK1	85.7	84.8	85.1	85.7	85.3	87.5	85.2	85.2	86.0	100.00	86.1	85.0	84.9	72.3	85.2
BCO1	97.6	87.6	87.1	97.0	94.4	87.2	94.3	94.1	94.2	86.1	100.00	89.4	87.9	72.4	97.1
KU2-6	89.5	89.8	90.0	89.5	89.7	88.6	89.5	89.6	89.0	85.0	89.4	100.00	90.8	72.5	89.3
Kn10	88.1	94.9	89.0	88.1	88.0	92.6	88.0	88.3	87.9	84.8	87.8	90.8	100.00	72.4	87.7
PS2	72.4	72.4	72.2	72.4	72.5	72.6	72.5	72.4	72.6	72.3	72.5	72.3	72.5	100.00	72.5
25	97.6	87.5	87.1	97.0	94.0	86.9	93.9	93.8	94.0	85.2	97.0	89.3	87.8	72.7	100.00

The ANI value (%) shared between genomes (above and below diagonal). AB04 ^T^ = *A. ayderensis* AB04^T^ [30]; WK1 = *A. flavithermus* WK1 [5, 29]; E13^T^ = *A. flavithermus* subsp. *yunnanensis* E13^T^ [35, 47, 48]; SK3-4 = *Anoxybacillus* sp. SK3-4 [45, 46]; DT3-1 = *Anoxybacillus* sp. DT3-1 [45, 46]; TNO = *A. flavithermus* TNO-09.006 [5, 44]; G10 = *A. kamchatkensis* G10 [40–42]; G2^T^ = *A. gonensis* G2^T^ [36]; AT ^T^ = *A. thermarum* AF/04^T^ [33–35]; AK1 = *A. flavithermus* AK1 [5, 39]; BCO1 = *Anoxybacillus* sp. BCO1 [31, 32]; KU2-6 = *Anoxybacillus* sp. KU2-6(11); Kn10 = *A. flavithermus* Kn10 [5, 43]; PS2 = *A. tepidamans* PS2 [37]; 25 = *A. flavithermus* 25 [5, 38]

### Analysis of the GHs in ***A. ayderensis*** AB04^T^ and other ***Anoxybacillus*** genomes

We detected 14 genes in the AB04^T^ genome encoding GH enzymes belonging to GH families 1, 10, 13, 31, 32, 51, 52, and 67 (Table 6). On average, the AB04^T^ GHs shared 93.9 % similarity with GHs identified in other *Anoxybacillus* spp. The GHs could be grouped into two types according to their predicted catalytic ability (Table 6). Nine GH enzymes were predicted to be active on α-chain polysaccharides whereas the remaining five GH enzymes were specific for β-linked polysaccharides (i.e., cellulose and xylan).

**Table 6:** List of several glycoside hydrolases (GHs) identified in various *Anoxybacillus* genomes

GH	Enzyme	Similarity within *Anoxybacillus* genome^a^														Number of studied enzyme^b^
		AB04^T^	WK1	E13^T^	SK3-4	DT3-1	TNO	G10	G2^T^	AF/04^T^	AK1	BCO1	KU2-6	Kn10	PS2	
1	β-glucosidase	91.2	100	90.7	39.5	92.4	-	-	-	-	-	-	91.6	92.0	-	-
10	Endo-1,4-β-xylanase	100.00	-	-	-	-	-	-	-	-	-	-	-	-	-	-
13	α-amylase (cell-bound)	98.6	100.00	98.4	97.4	96.2	97.2	96.2	98.2	98.6	94.5	76.6	98.8	99.8	84.3	2 [16]
13	α-amylase (extracellular)	77.6	-		100.00	-	-	95.0	-	-	-	54.2	-	-	-	-
13	Pullulanase	93.4	100.00	93.9	90.5	89.5	95.6	89.4	91.9	93.2	88.9	59.9	94.8	98.0	67.4	1 [17]
13	Amylopullulanase (>200 kDa)	-	100.00	90.2	88.8	-	-	99.1	-	-	59.6	-	-	89.6	-	1 [18]
13	Amylopullulanase (<200 kDa)	-	-	-	-	-	-	-	-	-	-	100	-	-	-	-
13	CDase	95.8	100.00	95.6	92.5	92.0	94.7	92.3	95.1	94.9	93.6	96.6	95.9	98.1	78.8	1 [19]
13	Oligo-1,6-glucosidase	98.2	100.00	61.7	96.0	96.0	98.1	96.0	53.9	97.5	97.0	53.6	96.5	98.6	90.9	-
13	Trehalose-6-phosphate hydrolase	95.6	100.00	-	94.2	93.7	-	93.9	-	96.2	94.9	-	95.8	99.1	-	-
13	1,4-α-glucan branching enzyme	93.4	100.00	-	93.2	92.8	94.1	92.4	-	93.9		94.9	-	-	-	-
31	α-glucosidase	92.1	100.00	92.9	91.0	89.2	88.1	89.2	91.5	91.7	88.5	71.1	93.4	96.9	67.4	-
32	Sucrase-6-phosphate hydrolase	94.7	100.00	-	91.3	91.8	91.0	91.3	-	93.5	92.5	-	93.9	93.3	-	-
36	α-galactosidase	-	-	91.2	-	-	-	79.2	100	-	90.5	72.3	93.7	-	79.5	-
51	α-L-arabinofuranosidase	93.6	100.00	-	-	-	-	-	-	-	99.4	-	-	-	-	1 [22]
52	β-xylosidase	91.5	-	90.4	-	-	-	100	99.6	-		-	-	-	89.5	-
65	Sugar hydrolase/phosphorylase	-	100.00	-	94.9	94.1	-	94.0		-	94.1	-	-	96.6	-	-
67	α-glucuronidase	100.00	-	-	-	-	-	-	-	-	-	-	-	-	-	-

^a^The reference for the protein sequence alignment is denoted as 100 %; ^b^The numbers represent the respective cloned, purified, and characterized enzymes from *Anoxybacillus* species. AB04^T^ = *A. ayderensis* AB04^T^ [30]; WK1 = *A. flavithermus* WK1 [5, 29]; E13^T^ = *A. flavithermus* subsp. *yunnanensis* E13^T^ [35, 47, 48]; SK3-4 = *Anoxybacillus* sp. SK3-4 [45, 46]; DT3-1 = *Anoxybacillus* sp. DT3-1 [45, 46]; TNO = *A. flavithermus* TNO-09.006 [5, 44]; G10 = *A. kamchatkensis* G10 [40–42]; G2^T^ = *A. gonensis* G2^T^ [36]; AT^T^ = *A. thermarum* AF/04^T^ [33–35]; AK1 = *A. flavithermus* AK1 [5, 39]; BCO1 = *Anoxybacillus* sp. BCO1 [31, 32]; KU2-6 = *Anoxybacillus* sp. KU2-6(11); Kn10 = *A. flavithermus* Kn10 [5, 43]; PS2 = *A. tepidamans* PS2 [37]

Interestingly, we found two GH enzymes that were uniquely present in strain AB04^T^: endo-1,4-β-xylanase (NCBI locus ID: KIP21668) and α-glucuronidase (KIP21917) (Table 6). The closest homologs of endo-1,4-β-xylanase and α-glucuronidase were found in *Geobacillus thermoglucosidans* and *Geobacillus stearothermophilus* with 81.9 % and 87.1 % sequence similarity, respectively [27].

Genes coding for at least five of the aforementioned GHs including cell-bound α-amylase, pullulanase, CDase, oligo-1,6-glucosidase, and α-glucosidase were consistently found in the genomes of all *Anoxybacillus* spp. (Table 6). Therefore, these enzymes might play an important role in *Anoxybacillus* carbohydrate metabolism. A high molecular-mass amylopullulanase (>200 kDa) from *Anoxybacillus* sp. SK3-4 has been reported previously [18]. We detected this enzyme in other *Anoxybacillus* spp., for instance *A. flavithermus* WK1 [5, 29], *A. flavithermus* subsp. *yunnanensis* E13^T^ [35, 47, 48], *A. kamchatkensis* G10 [40–42], *A. flavithermus* AK1 [5, 39], and *A. flavithermus* Kn10 [5, 43]. From the current analysis, it can be concluded that amylopullulanase is the GH with greatest molecular-mass in *Anoxybacillus* (Table 6). Despite their widespread distribution in *Anoxybacillus* spp*.*, only a limited number of GHs have been studied intensively. At present, only α-amylase [16], pullulanase [17], amylopullulanase [18], CDase [19], and α-L-arabinofuranosidase [22] have been cloned, purified, and biochemically characterized (Table 6). he number of underexplored GH enzymes such as β-glucosidase, endo-1,4-β-xylanase, α-L-arabinofuranosidase, α-glucuronidase, and β-xylosidase remains high; however, because of their interesting applications and their important roles in second-generation biofuel production [69], these enzymes are worthy of examination in the near future.

### Other ***A. ayderensis*** AB04^T^ enzymes with potential applications

Apart from the GHs, we found that *A. ayderensis* AB04^T^ has genes coding for other industrially important enzymes such as xylose isomerase, esterase, and aldolase. Xylose isomerase (EC 5.3.1.5) catalyzes the isomerization of xylose to xylulose and of glucose to fructose, which is important in the industrial production of high-fructose corn syrup [20]. Earlier, a xylose isomerase from *A. gonensis* G2^T^ was characterized and the enzyme displays 96.8 % amino acid sequence similarity to the one identified in strain AB04^T^ (KIP21927) [20].

Previous studies have indicated that *A. gonensis* G2^T^, *A. gonensis* A4, and *Anoxybacillus* sp. PDF-1 produce esterase [70–72]. We identified two esterases (KIP19922 and KIP21735) in the genome of strain AB04^T^, which shared 96.3 % and 96.0 % amino acid sequence similarity with the esterase from *Anoxybacillus* sp. PDF-1 [72] and *A. gonensis* G2^T^ [70], respectively. In addition, a fructose-1,6-bisphosphate aldolase from *A. gonensis* G2^T^ has been described [73]. Strain AB04^T^ carries two aldolases, KIP21451 and KIP21450, which showed 95.9 % and 99.9 % amino acid similarity to aldolase from *A. flavithermus* WK1 [5, 29] and *A. thermarum* AF/04^T^ [33–35], respectively. We did not biochemically characterize these enzymes from strain AB04^T^ in the current study.

Thermophilic bacteria are highly sought after for their potential use in bioremediation processes. Several *Anoxybacillus* spp. efficiently reduce metal ions such as Hg^2+^, Cr^4+^,Al^3+^, and As^3+^ [4, 23–25]. The genome of strain AB04^T^ contains at least six heavy metal resistance genes. Four genes are related to mercuric ion reduction; two of these are mercury resistance (*mer*) operons (KIP20706 and KIP20408) and the two other genes encode mercuric reductases, which catalyze the reduction of Hg^2+^ to Hg^0^ (KIP19952 and KIP20409). In addition, strain AB04^T^ carries genes for an arsenate reductase (KIP20402) and an arsenic efflux pump protein (KIP20401). The function of these genes will be studied in the close future.

## Conclusions

Knowledge on the genomics, industrial enzymes, and relevant applications of *Anoxybacillus* spp. are rather limited compared to that in their closest relatives, *Geobacillus* and *Bacillus*. In the present work we presented a whole-genome sequence of *A. ayderensis* AB04^T^ and its annotation. Additionally, we provided insights into several GHs, under-explored enzymes, and putative applications of strain AB04^T^.

## Abbreviations

ANI: Average Nucleotide Identity
CDase: Cyclomaltodextrinase
FAME: Fatty acid methyl esters
FESEM: Field emission scanning electron microscope
GH: Glycoside hydrolase
ML: Maximum-likelihood
